# Psychological Resources are Associated with Reduced Incidence of Coronary Heart Disease. An 8-Year Follow-up of a Community-Based Swedish Sample

**DOI:** 10.1007/s12529-014-9387-5

**Published:** 2014-01-16

**Authors:** Oskar Lundgren, Peter Garvin, Lena Jonasson, Gerhard Andersson, Margareta Kristenson

**Affiliations:** 1 Department of Medical and Health Sciences, Division of Community Medicine, Linköping University, Linköping, 595 83 Sweden; 2 Department of Medical and Health Sciences, Division of Cardiovascular Medicine, Linköping University, Linköping, Sweden; 3 Department of Behavioural Sciences and Learning, Swedish Institute for Disability Research, Linköping University, Linköping, Sweden; 4 Department of Clinical Neuroscience, Section of Psychiatry, Karolinska Institute, Stockholm, Sweden

**Keywords:** Mastery, Self-esteem, Sense of coherence, Hopelessness, Psychosocial, Coronary heart disease, Myocardial infarction

## Abstract

**Background:**

A large number of studies have provided clear evidence for a link between the risk of coronary heart disease and psychological risk factors. Much less attention has been given to the potential protective effect of psychological resources.

**Purpose:**

The major aim of this study was to investigate the independent association between psychological resources and incidence of coronary heart disease (CHD) in an 8-year follow-up study of a Swedish community-based cohort.

**Methods:**

The cohort consisted of 484 men and 497 women, aged 45–69 years at baseline. The incidence of first-time major event of CHD was analysed in relation to baseline levels of psychological resources, including mastery, self-esteem, and sense of coherence as well as psychological risk factors including cynicism and hostile affect, vital exhaustion, hopelessness, and depressive symptoms. In Cox proportional hazard models, adjustments were made for age, sex, eight traditional cardiovascular risk factors, and depressive symptoms.

**Results:**

A total of 56 CHD events had occurred after the 8-year follow-up. After adjustment for age, sex, and eight traditional risk factors, a significantly decreased risk of CHD was found for mastery (HR 0.62 per SD, *p* = 0.003), self-esteem (HR 0.64, *p* = 0.004), and sense of coherence (HR 0.70, *p* = 0.031). An increased risk of CHD was found for vital exhaustion (HR 1.46, *p* = 0.014), hopelessness (HR 1.59, *p* = 0.003), and depressive symptoms (HR 1.45, *p* = 0.009). After further adjustment for depressive symptoms, significant associations remained for mastery (HR 0.67, *p* = 0.034), self-esteem (HR 0.69, *p* = 0.048), and hopelessness (HR 1.48, *p* = 0.023).

**Conclusions:**

The psychological resources, mastery and self-esteem, showed robust protective effects on CHD, also after adjustment for established risk factors as well as depressive symptoms. In parallel, hopelessness was an independent risk factor for CHD. The results may have implications for novel approaches in preventive efforts.

## Introduction

Since the 1940s, a continually growing interest in the relationship between cardiovascular disease and psychological distress has generated a rich amount of scientific data [1], with solid evidence for significant associations between risk of coronary heart disease (CHD) and psychological risk factors like perceived stress [2, 3], anxiety [3], hostile affect [3, 4], vital exhaustion [5], hopelessness [6], and depression [3, 7]. Large case–control studies have shown that psychological risk factors can have equal impact on risk for myocardial infarction as traditional risk factors, such as smoking, dyslipidaemia, and high blood pressure [8, 9]. However, much less attention has been given to the potential protective effect of psychological resources [10]. In recent years, a handful of prospective studies have reported a protective effect of mastery, sense of coherence [11], and optimism [12] on coronary heart mortality.

Two earlier studies have prospectively linked psychological resources with decreased incidence of CHD [13, 14]. One of those was a 12-year follow-up of 88,175 men and women in Japan showing that a single-item measure of life enjoyment was associated with a lower incidence of CHD for men but not for women [13]. The other was a 15-year follow-up of 6,026 men and women in the USA, demonstrating that high scores for a measure of emotional vitality reduced the risk of incident CHD [14]. Prospective studies including reliable psychological measures of both psychological resources and risk factors are scarce [11, 12, 14].

The major aim of this study was to investigate the independent association between measures of psychosocial resources and incidence of CHD in an 8-year follow-up study of a Swedish community-based cohort. In addition, we examined the effects of established psychological risk factors.

## Methods

### Study Population & Procedures

The longitudinal study “Life conditions, Stress and Health” (LSH) aims to investigate to what extent psychosocial characteristics can explain socioeconomic differences in the risk of CHD, and if psychobiological pathways can mediate these associations. The study population is a random sample of 499 men and 502 women aged 45–69 years. The study was performed in collaboration with 10 primary health care centres in the County of Östergötland in South East Sweden. Participants were invited consecutively to reach a study population size of *n* = 1,000, evenly distributed by age and sex. All citizens in the given age range living in any of the catchment areas of these 10 primary health care canters in the County of Östergötland in South East Sweden at the time of enrolment were eligible for invitation. The procedure was based on the methodology used in the HAPIEE-study [15]. The response rate was 62.6 %, corresponding to 1,628 invited participants.

Exclusion criteria were serious physical conditions (e.g., terminal cancer) or psychiatric illnesses that would interfere with practical procedures (e.g., psychosis). One participant was excluded due to a diagnosed terminal pancreatic cancer. Overall, the sample was representative for the population in terms of educational attainment, employment rates, and immigrant status.

Initial data collection was conducted in late 2003 and early 2004. Participants filled out a set of questionnaires, covering demographic information, socioeconomic status, psychosocial factors, lifestyle, and previous and present diseases. The regional ethical review board, Linköping University, approved the study design, and informed consent was obtained from all participants.

### Psychological Resources

The psychological resources examined were chosen to capture constructs that are viewed as intrinsic factors, constituting a buffering capacity against stressors [16–18]. Sense of mastery captures feelings of confidence and self-reliance with an experience that life to some extent is manageable and was measured with Pearlin and Schooler's 7-item questionnaire [16]. The degree of self-esteem was measured with Rosenberg's 10-item questionnaire [17], which contains both a global dimension of basic self-worth and a comparison with other people's competences. Sense of coherence in life was measured with Antonovsky's 13-item version [18]. The construct of coherence is a three-dimensional construct that captures an experience of life as being comprehensible, manageable, and meaningful. The scales are all validated and extensively used, with robust findings in earlier studies of intermediate outcomes such as inflammatory biomarkers and cortisol [19, 20], as well as hard outcomes such as CHD and mortality [11, 21].

### Psychological Risk Factors

All selected risk factors, assessed by validated instruments, are known to be risk factors for CHD [3–7]. Symptoms of depression were measured with the 20-item questionnaire from the Centre for Epidemiological Studies (CES-D) [22]. This scale has a cut-off for (mild) clinical depression at 16 points. Vital exhaustion was measured with a 19-item questionnaire that captures feelings of physical and mental fatigue [23]. The experience of hopelessness was measured with a 2-item questionnaire [6]. Two subscales of the Hostility scale were used; the extent to which subjects felt cynical distrust (cynicism) and hostile affect in relationship to other people was measured with a 12-item and a 5-item scale, respectively [24].

### Measures of Behavioural and Traditional Risk Factors

The questionnaire included items about smoking habits. Current smokers and those who had stopped smoking in the last 5 years were defined as smokers. Physical activity was assessed by a combination of structured exercise and unstructured physical activity, and four groups were created according to health guidelines [25]. Questions regarding consumption of fruit and vegetables were derived from the validated Food Frequency Questionnaire adopted from the Swedish Mammography Cohort [26], and data were dichotomized into the categories above or below 500 g/week, corresponding to the Swedish standard of recommended daily intake. Questions on alcohol consumption were adopted from the same questionnaire [26]. The five groups of alcohol intake were defined as no intake (0 g/week) or low to moderate (0.1–80 g/week), high (81–160 g/week), and very high (>160 g/week) intake, and cut-offs were guided by earlier studies of cardiovascular benefits and risk associated with drinking [27].

During a visit to one of the 10 collaborating primary health care (PHC) centres, participants underwent a short vital health status examination, including height, weight, and blood pressure; the latter being measured in a sitting position in 2 min interval after 5 min rest, using the mean of the second and third measurements (Omron M5-1 digital). Blood samples were collected in a fasting state. Plasma lipids were measured directly after sample collection (with an ADVIA 1650 analyser), and LDL-cholesterol was calculated using Friedewald's formula [28]. Self-reported presence of diabetes mellitus was based on the question “Have you ever been diagnosed with diabetes by a physician?” (yes/no).

### Follow-up of Cardiovascular Outcomes

Main outcome was first-time major event of CHD, defined as fatal or nonfatal myocardial infarction and/or an event of invasive coronary revascularisation, defined as percutaneous cardiac intervention or coronary artery by-pass graft-surgery. Outcome data after 8 years of follow-up was obtained from the Cause of Death Registry and the Registry of Hospital Admissions (covering more than 99 % of hospital discharges), both from the Swedish National Board of Health and Welfare. The events and causes of death were further cross-validated using the patients’ medical reports.

### Statistical Analysis

First-time incidence of CHD was set as the main outcome. First day of follow-up for each participant was defined as the date of PHC visit. Last day of follow-up was set to 31 Dec 2011, and follow-up time was truncated by CHD events or any fatal events. Participants with a prior history of myocardial infarctions, based on the response to the question: do you have any of the following diseases as diagnosed by a physician (yes/no), were excluded from further analyses (*n* = 20). The same question gave information about the prevalence of diabetes mellitus.

Spearman correlation coefficients were used to display how the psychological measures were related to each other. A set of Cox proportional hazard models with CHD incidence as outcome was defined for each psychosocial variable, adding potential confounders in three steps. The first step included adjustment for age and sex, the second step age, sex and smoking, physical activity, alcohol intake, fruit and vegetable intake, blood-pressure, lipid levels, BMI, and diabetes mellitus. The third step included all the above factors plus depressive symptoms (CES-D).

In this third step, depressive symptoms were included in two ways; as a dichotomous variable using a CES-D cut-off score of 16 or higher (since this has been suggested to discriminate clinically significant depressive symptoms in a general population [22] or as a continuous score of CES-D). A two-sided probability value of *p* ≤ 0.05 was considered as statistically significant. Analyses were performed in STATA statistical software, release 11.0 (Stata Corporation, College Station, TX, USA) and IBM SPSS for Windows statistical software, release 21 (IBM Corporation, Armonk, NY, USA).

## Results

Fifty-six new cases of CHD were identified over 7,502 person years (*n* = 981). This corresponds to a mean incidence of 7.5 cases per 1,000 persons per year and a mean follow-up time of 7.6 years per participant (range 0.7 to 8.2 years).

The demographic characteristics of the study population are shown in Table 1 and results of psychological measurements are shown in Table 2. Descriptive analyses showed lower scores on resources and higher scores on risk factors for women, but small differences for age (data not shown). Correlation coefficients between psychological measures and the covariates in Table 1 (cardiovascular risk factors) were small (*r* < 0.2 for all).

**Table 1:** Descriptive and demographic characteristics

	*n* (%)	Mean (SD)
Age	981 (100)	57 (7.1)
Sex (male/female)	484/497 (49/51)	–
Behavioural factors		
Smoking	948 (97)	
Yes	208 (22)	–
No	740 (78)	–
Physical activity^a^	908 (93)	
Hardly any (lowest)	39 (5)	–
Light activity (2nd)	302 (33)	–
Moderate activity (3rd)	394 (43)	–
Hard activity (highest)	173 (19)	–
Alcohol intake^b^	959 (98)	
None	57 (6)	–
Low to moderate (0–80 g/week)	721 (75)	–
High (81–160 g/week)	92 (10)	–
Very high (>160 g/week)	71 (6)	–
Quit	18 (2)	–
Fruit and vegetable intake^c^	962 (98)	
Low	474 (48)	–
High	508 (52)	–
Laboratory characteristics		
Systolic blood pressure (mmHg)	968 (99)	134 (20.0)
Diastolic blood pressure (mmHg)	968 (99)	85 (11.5)
LDL cholesterol (mmol/L)	957 (98)	3.5 (0.9)
HDL cholesterol (mmol/L)	973 (99)	1.6 (0.4)
Triglycerides (mmol/L)	973 (99)	1.4 (0.9)
Anthropometric measures		
Body mass index (kg/m^2^)	972 (99)	26.8 (4.3)
Waist circumference (cm)	976 (99)	92.0 (12.4)

^a^Categories based on a combination of leisure-time and physical activity at work

^b^Gram/week, cut-offs according to risk levels

^c^Gram/day, cut-off >500 g, according to recommended intake

**Table 2:** Characteristics of psychological measures

Psychological measure	Number of items	Cronbachs’ alpha	Range in instrument	Range in study pop	Mean (SD)	Median (IQR)
Mastery (*n* = 924)	7	0.75	7–28	7–28	22.6 (3.4)	23 (20;25)
Self-esteem (*n* = 923)	10	0.85	10–40	15–40	32.2 (4.8)	33 (30;36)
Sense of coherence (*n* = 938)	13	0.81	13–91	32–91	68.7 (10.4)	70 (62;77)
Cynicism (*n* = 945)	12	0.85	12–60	12–53	31.3 (7.7)	31 (26;36)
Hostile affect (*n* = 943)	5	0.44	5–25	5–23	11.6 (2.9)	12 (9;14)
Vital exhaustion (*n* = 941)	19	0.93	19–57	19–56	30.2 (7.6)	29 (24;35)
Hopelessness (*n* = 939)	2	0.69	0–8	0–8	2.3 (2.0)	2 (1;4)
Depressive symptoms, CES-D (*n* = 913)	20	0.86	0–60	0–51	9.0 (7.9)	7 (3;12)

As presented in Table 3, the correlations between different psychological measures were generally high but with a large variation, ranging from *r* = 0.13 (cynicism and vital exhaustion) to *r* = 0.68 (mastery and self-esteem). Hostile affect and cynicism scales were correlated to each other, *r* = 0.47, but showed weaker albeit significant correlations with other psychological measures (all *p* < 0.01).

**Table 3:** Intercorrelational matrix for psychological measures

	A	B	C	D	E	F	G	H	I
Mastery A	1	–	–	–	–	–	–	–	–
Self-esteem B	0.68	1	–	–	–	–	–	–	–
Sense of coherence C	0.56	0.58	1	–	–	–	–	–	–
Cynicism D	−0.16	−0.18	−0.32	1	–	–	–	–	–
Hostile affect E	−0.28	−0.25	−0.45	0.47	1	–	–	–	–
Vital exhaustion F	−0.54	−0.56	−0.61	0.13	0.25	1.	–	–	–
Hopelessness G	−0.58	−0.51	−0.44	0.27	0.25	43	1	–	–
Depressive symptoms H	−0.51	−0.52	−0.54	0.15	0.24	0.67	0.46	1	–
Depressive symptoms dichotomous (>16) I	−0.43	−0.42	−0.45	0.12	0.20	0.56	0.38	0.86	1

Spearman coefficients, (*n* = 875). All correlation coefficients are significant, *p* < 0.01

Findings from the Cox proportional hazard models are presented in Table 4. In the full models, total time-at-risk ranged from 5,975 to 6,141 person years and from 39 to 41 new events, depending on psychological measure evaluated and numbers of participants without missing data.

**Table 4:** Cox-proportional hazard model adjusted for age, sex, and cardiovascular risk factors

Psychological factors	Model a			Model b			Model c		
	HR	CI (95 %)	*p*-Value	HR	CI (95 %)	*p*-Value	HR	CI (95 %)	*p*-Value
Mastery (*n* = 781)	0.62	0.47–0.82	0.001	0.62	0.46–0.85	0.003	0.67	0.46–0.97	0.034
Self-esteem (*n* = 777)	0.64	0.48–0.86	0.003	0.64	0.47–0.86	0.004	0.69	0.47–1.00	0.048
Sense of coherence (*n* = 796)	0.70	0.52–0.95	0.023	0.70	0.51–0.96	0.031	0.78	0.55–1.15	0.217
Cynicism (*n* = 800)	1.04	0.76–1.43	0.777	0.96	0.68–1.36	0.851	0.92	0.64–1.31	0.655
Hostile affect (*n* = 796)	1.12	0.84–1.52	0.449	1.11	0.81–1.53	0.516	1.06	0.76–1.48	0.715
Vital exhaustion (*n* = 799)	1.56	1.16–2.08	0.003	1.46	1.07–1.97	0.014	1.34	0.88–2.03	0.169
Hopelessness (*n* = 794)	1.56	1.15–2.11	0.003	1.59	1.16–2.17	0.003	1.48	1.06–2.08	0.023
Depressive symptoms (*n* = 803)	1.58	1.20–2.08	0.001	1.45	1.09–1.92	0.009	1.40	0.85–2.27	0.185
Depressive symptoms (*n* = 803) (dichotomy)	2.47	1.21–5.04	0.001	2.31	1.09–4.91	0.028	–	–	–

Hazard ratio expressed per SD increment except for depressive symptoms, defined as a dichotomy based on 16 or above on CES-D score. Models adjusted for a) age and sex, b) age, sex, diabetes, BMI, blood pressure, blood lipids, smoking, physical inactivity, high alcohol intake, and low fruit & vegetables intake, and c) as model b plus depressive symptoms (CES-D ≥16)

After adjustment for age and sex, significant protective effects against CHD were found for psychological resources: mastery (HR = 0.62 per SD), self-esteem (HR = 0.64), and sense of coherence (HR = 0.70). Depressive symptoms were significantly associated with increased risk of CHD regardless of evaluating a dichotomy of CES-D score (HR = 2.47 per SD), or a continuous CES-D score (HR = 1.58 per SD). Likewise, vital exhaustion (HR = 1.56) and hopelessness (HR = 1.56 per SD) were also associated with a significantly increased risk of CHD. All these associations remained statistically significant after adjustment for cardiovascular risk factors. In the adjusted models, smoking, lipid levels, and blood pressure remained positively associated with the risk of CHD, along with any of the psychological factors (data not shown). Scale scores of cynicism and hostile affect showed no significant relationships with the incidence of CHD, regardless of adjustment.

After adjustment for age, sex, eight traditional cardiovascular risk factors, and depressive symptoms (using 16 as a cut-off), mastery and self-esteem still displayed significant protective effects (HR = 0.67 per SD, *p* = 0.034 and HR = 0.69 per SD, *p* = 0.048, respectively). Moreover, hopelessness remained significantly associated with increased CHD incidence (HR = 1.48 per SD, *p* = 0.023).

Repeating these models using CES-D as a continuous score yielded marginal significant association for mastery (*p* = 0.064) and self-esteem (*p* = 0.098), whereas hopelessness remained significant (*p* = 0.047).

To address a potential interaction effect of hopelessness and self-esteem, we performed a post-hoc analysis running self-esteem and hopelessness in the same model along with age, sex, and cardiovascular risk factors. This showed that self-esteem remained significant (*p* = 0.031), whereas hopelessness did not (*p* = 0.210).

The independent effect of depressive symptoms, entered as either dichotomous or continuous variable, was lost in all models when entered together with mastery, self-esteem, or hopelessness, respectively (*p*-value for CES-D ranging from *p* = 0.201 to *p* = 0.520). Using antidepressant medication instead of CES-D in model c, as a means to adjust for a potential confounding factor, did not affect the results.

## Discussion

The main finding in this study was that mastery, self-esteem, and sense of coherence were significantly associated with a reduced risk of CHD, in a model adjusted for age, sex, and eight cardiovascular risk factors. It is noteworthy that adjustment for conventional physiological and health behavioural risk factors did not affect the independent effect of psychological resources, since health behaviour has been proposed as a pathway that mediates the association between positive psychological factors and health outcome [10, 14]. Moreover, mastery and self-esteem remained significantly associated with a reduced risk of CHD when depressive symptoms were entered into the model.

To our knowledge, this is the first prospective study demonstrating the independent protective effect of mastery and self-esteem on incident CHD. The findings on mastery are in line with one previous study, where mastery was linked to cardiovascular mortality after adjustment for smoking and the psychological risk factors hostility and neuroticism, in a 6-year follow-up of 20,323 men and women in the UK [11]. The findings on self-esteem are partly in line with results from a 5-year follow-up study of 2,682 men in Finland, where a protective effect of high self-esteem on all-cause mortality was demonstrated, but lost statistical significance after adjustment for the psychological risk factors hopelessness, depression, cynical hostility, and sullenness [21]. In particular, these authors highlight hopelessness as a substantial confounder for the relation between self-esteem and total mortality. Therefore, we performed a post-hoc analysis running self-esteem and hopelessness in the same model along with age, sex, and cardiovascular risk factors. In that model, self-esteem remained significant (*p* = 0.031), whereas hopelessness did not (*p* = 0.210).

Furthermore, our findings are in line with a 15-year follow-up of 887 men in the Netherlands, where dispositional optimism was found to be associated with reduced cardiovascular mortality, after adjustment for traditional cardiovascular risk factors and depressive symptoms [12].

Our results for vital exhaustion, hopelessness, and depressive symptoms corroborate findings from several other studies [5–7, 21]. Previous studies of hostility are more inconclusive, researchers arguing both for [4, 29] and against [30] a link between hostility and CHD. Our findings do not support that hostility, determined by either cynicism or hostile affect, is associated with risk of CHD.

The relatively strong inverse correlations between psychological resources and depressive symptoms (Table 3) may imply overlap between constructs, calling for nuanced interpretations. However, it is also possible that psychological resources and depressive symptoms are related to each other, which make it equally important to not routinely make adjustment for their independent effects. Interestingly, both mastery and self-esteem (but not sense of coherence) remained significantly associated with incidence of CHD after controlling for depressive symptoms. Moreover, the association between depressive symptoms and CHD risk became nonsignificant in these three models.

The results bring further support to a potential cardioprotective effect of positive psychological attributes [31]. The items in the mastery questionnaire reflect a hopeful view that life is filled with possibilities and directions and, also, that problems are manageable. It is basically a measure of trust in one’s own resources, competence, and ability to cope with difficulties [16]. Likewise, the measure of self-esteem aims to capture an attitude of positiveness towards oneself, as well as sense of self-worth [16]. Over time, it is possible that the presence of a positive outlook and adaptive coping strategies are building psychological, physical, and social resources in an upward spiral [32]. This may lead to resilience and flexibility of the stress response, being important for the maintenance of a dynamic balance in and between autonomic [33], neuroendocrine [19, 34, 35], and neuroimmunologic [20, 33] systems. A dysregulation of these systems may allow a state of chronic low-grade inflammation to occur and, thereby, also constitute a link to CHD [10]. Accordingly, we showed in recent cross-sectional studies that both mastery and self-esteem were inversely associated with circulating markers of inflammation and atherosclerotic plaque vulnerability [20, 36].

In the present study, we also demonstrated that hopelessness remained significantly associated with the incidence of CHD after adjustment for depressive symptoms, implying an independent association. This finding is interesting since hopelessness is one of the symptoms in the clinical presentation of depression [37]. The effects of different depressive symptoms on CHD risk deserve further study, and it could be that hopelessness is a stronger risk factor, at least for CHD, than other symptoms of depression. Indeed, in a recent study, we found that both depression and hopelessness were associated with systemic inflammation, assessed by interleukin-6 in plasma, and this effect remained for hopelessness after adjustment for depressive symptoms, but not for depression after adjustment for hopelessness [38].

### Strengths and Limitations

There are several strengths of our study. We used eight validated and reliable measures of both positive and negative psychological factors in a well-characterised community-based cohort. The validity of the outcome is strengthened by the results showing that the traditional established cardiovascular risk factors, i.e., smoking, dyslipidaemia and blood pressure, turned out to be significant risk factors in all models. The validity of our data is further supported by the consistency in regression models showing that different psychological resources were significantly associated with CHD during follow-up.

The incidence rate for major CHD events was, in this study, 7.5 cases per 1,000. This was lower than the national average in Sweden (9.6 per 1,000) in this age group during the same time period [39]. Moreover, the mean scale score of mastery (22.6 vs. 22.6) [16] was the same as in another Swedish sample of a middle-aged population, and mean levels of depressive symptoms (9.0 vs. 9.3) [22], and hopelessness (2.3 vs. 2.7) [6], were slightly lower in our study when compared with similar samples. Taken together, this indicates that our study population is somewhat healthier than the overall population. This could be viewed as strength since our findings were significant and robust despite a risk of healthy selection bias.

One major limitation, on the other hand, is the relatively low number of cases during follow-up. Due to missing data of at least one adjustment factor, this limitation may be further accentuated in full regression models thus opening up for type II errors. Our choice of variables, adjusted for in the regression models, is based on what is known to be of major importance for CHD risk [8, 9].

### Implications for Clinical Practice

Increased knowledge on the protective effect of psychological resources might have a great impact on both primary and secondary preventive efforts. Novel approaches in this domain are especially called for since attempts to treat depression in CHD patients with pharmacological or psychological interventions have been less successful so far [40–42]. One alternative may be to build and cultivate psychological resources instead of focusing on psychological distress. Interestingly, early attempts to intervene by cultivating psychological resources have revealed promising results in clinical studies [43–47]. The results of our study may highlight the need of new screening tools for identifying CHD patients with a lack of psychological resources [48, 49]. Furthermore, an increased awareness of cardioprotective psychological resources may be important for public health practitioners and policy makers.

## Conclusions

The psychological resources mastery and self-esteem showed robust protective effects on CHD, also after adjustment for established risk factors as well as depressive symptoms. In parallel, hopelessness was an independent risk factor for CHD. The results may have implications for novel approaches in preventive efforts.
